# Deep learning for the fully automated segmentation of the inner ear on MRI

**DOI:** 10.1038/s41598-021-82289-y

**Published:** 2021-02-03

**Authors:** Akshayaa Vaidyanathan, Marly F. J. A. van der Lubbe, Ralph T. H. Leijenaar, Marc van Hoof, Fadila Zerka, Benjamin Miraglio, Sergey Primakov, Alida A. Postma, Tjasse D. Bruintjes, Monique A. L. Bilderbeek, Hammer Sebastiaan, Patrick F. M. Dammeijer, Vincent van Rompaey, Henry C. Woodruff, Wim Vos, Seán Walsh, Raymond van de Berg, Philippe Lambin

**Affiliations:** 1 The D-Lab, Department of Precision Medicine, GROW-School for Oncology, Maastricht University, Maastricht, The Netherlands; 2 Department of Radiology and Nuclear Imaging, GROW-School for Oncology, Maastricht University Medical Centre, Maastricht, The Netherlands; 3 Oncoradiomics SA, Clos Chanmurly 13, 4000 Liège, Belgium; 4 Department of Otolaryngology and Head and Neck Surgery, Maastricht University Medical Center, Maastricht, The Netherlands; 5 Department of Radiology and Nuclear Medicine, Maastricht University Medical Center, Maastricht, The Netherlands; 6 School for Mental Health and Sciences, Maastricht University, Maastricht, The Netherlands; 7 Department of Otorhinolaryngology, Gelre Hospital, Apeldoorn, The Netherlands; 8 Department of Otorhinolaryngology, Leiden University Medical Center, Leiden, The Netherlands; 9 Department of Radiology, Viecuri Medical Center, Venlo, The Netherlands; 10 Department of Otorhinolaryngology, Viecuri Medical Center, Venlo, The Netherlands; 11 Department of Otorhinolaryngology and Head & Neck Surgery, Antwerp University Hospital, Antwerp, Belgium; 12 Department Translational Neuroscience, Faculty of Medicine and Health Sciences, University of Antwerp, Antwerp, Belgium; 13 Department of Radiology and Nuclear Medicine, GROW-School for Oncology and Developmental Biology, Maastricht University Medical Centre+, Maastricht, The Netherlands; 14 Haga Hospital, Radiology, Els Borst-Eilersplein 275, Den Haag, Zuid-Holland The Netherlands

**Keywords:** Image processing, Mathematics and computing, Computer science

## Abstract

Segmentation of anatomical structures is valuable in a variety of tasks, including 3D visualization, surgical planning, and quantitative image analysis. Manual segmentation is time-consuming and deals with intra and inter-observer variability. To develop a deep-learning approach for the fully automated segmentation of the inner ear in MRI, a 3D U-net was trained on 944 MRI scans with manually segmented inner ears as reference standard. The model was validated on an independent, multicentric dataset consisting of 177 MRI scans from three different centers. The model was also evaluated on a clinical validation set containing eight MRI scans with severe changes in the morphology of the labyrinth. The 3D U-net model showed precise Dice Similarity Coefficient scores (mean DSC-0.8790) with a high True Positive Rate (91.5%) and low False Discovery Rate and False Negative Rates (14.8% and 8.49% respectively) across images from three different centers. The model proved to perform well with a DSC of 0.8768 on the clinical validation dataset. The proposed auto-segmentation model is equivalent to human readers and is a reliable, consistent, and efficient method for inner ear segmentation, which can be used in a variety of clinical applications such as surgical planning and quantitative image analysis.

## Introduction

The inner ear, also known as the labyrinth, is a complex structure located in the temporal bone. It roughly consists of the cochlea, the vestibule, and the semi-circular canals. Understanding changes and variations within these structures can help diagnose and predict a number of conditions^1^, such as inflammatory and neoplastic processes. Technological developments in imaging techniques have allowed (neuro)radiologists to evaluate the human labyrinth, with recent advances increasing the level of detail^1^. Moreover, applications of artificial intelligence and the quantitative assessment of medical images for the non-invasive exploration of anatomical structures and the classification of diseases have remarkably increased in recent years^2^.

The process of the automated extraction and analysis of large amounts of quantitative information from medical images is known as *radiomics*^3,4^. A recent study investigated the value of *radiomics* for diagnosis of Meniere’s disease (MD), an inner ear disorder characterized by episodic vertigo spells, hearing loss, and tinnitus^5^. Other labyrinthine disorders such as sensorineural hearing loss might benefit from quantitative image analysis as well^6^.

Image segmentation is a critical step to work towards fully automated diagnostic tools for inner ear disorders. Manual segmentation requires experienced readers, is time-consuming and prone to intra-and inter-observer variability^7–9^. Over the past years, several automatic and semi-automatic inner ear segmentation methods were proposed for both MRI and CT imaging^10–14^, including region-growing, thresholding and edge detection^15^, model-based^10,14^, atlas-based^12,13^ and machine-learning techniques^11^. The inner ear’s small and complex structure makes segmentation challenging, especially in MR imaging due to non-homogenous image intensities^10,11^.

Recent work proposed a statistic shape model (SSM) for inner ear segmentation in MR images (13). However, the proposed methodology present a high computational burden, both in term of time and cost. Another recently published segmentation model showed very good agreement between an atlas-based segmentation and the manual gold standard^12^, yet requires manual intervention. Additionally, segmentation performance of atlas-based methods decreases for complex structures with variable shape and size^16^.

Recent studies have demonstrated the successful application of deep learning techniques for detection, segmentation, and classification tasks in the medical field^17^. Among deep learning techniques, the U-Net architecture is a specific type of convolutional neural network (CNN) consisting of multilayer neural networks. These networks have been implemented successfully, especially for auto-segmentation in medical images^18,19^. Although U-Net based deep learning approaches do exist for segmentation of the inner ear^18,20^, they lack the incorporation of anatomical variations, pathological situations or missing anatomical structures, which are part of daily clinical practice. Hence, there is currently no fully automated, generic segmentation method for the inner ear to meet the growing demand for the developments in 3D visualization and quantitative image assessments.

Therefore, this study’s objective was to develop a deep-learning approach for the automatic segmentation of the inner ear in clinical MR images, focusing on the robustness of the method in varying clinical situations, and to evaluate its performance and generalizability with manual segmentation as reference.

## Materials and methods

### Ethical considerations

This study was performed in accordance with the guidelines outlined by Dutch and Belgian legislation. MRI scans were collected and fully anonymized by the local investigators of four centers. Ethics committee of University Hospital Antwerp approved the study (Approval number—17/09/093) and written informed consent was obtained from the participants. The other centers waived the ethics approval due to the retrospective nature and full anonymization of the data according to the Medical Research involving Human Subjects act (WMO).

### Automatic segmentation workflow

**Figure 1:**
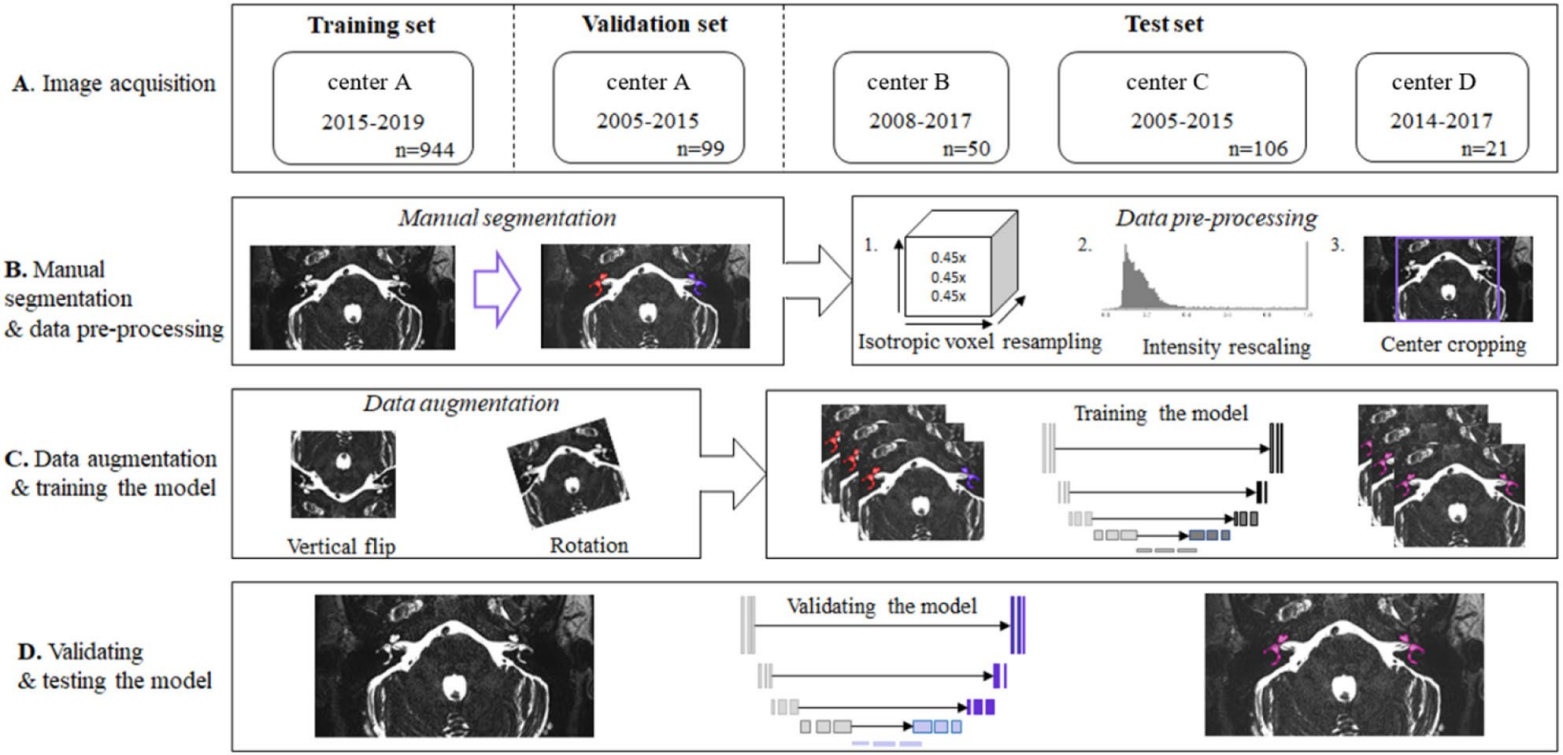
The workflow of autosegmentation of the inner ear in this study, graphically presented in four steps. (**A**) The image acquisition from four different centers divided into training, validation and an independent test set. (**B**) Manual segmentation of the labyrinth and pre-processing steps consisting of isotropic voxel resampling, intensity rescaling and center cropping. (**C**) Extending the data set (data augmentation) by flipping and rotating the input images and training of the model. (**D**) Validation and testing the model on an independent test cohort.

The workflow applied in this study consisted of four steps and is illustrated in Fig. 1. Each step of the workflow is detailed in the following paragraphs. Additional details on the model architecture, training, validation and testing can be found in Supplementary Sect. 1 of the Supplementary Information.

### Training dataset

A total of 1203 images of patients who underwent an MRI scan of the cerebellopontine angle for diverse neuro-otological indications in the period of December 2015 to April 2019 in Maastricht Medical University center (center A) were collected and fully anonymized. All high resolution T2-weighted images were acquired in 1.5 and 3 T (T) MRI scanners, from different vendors with a variety of high-resolution T2-weigted sequences (3D cochlea, DRIVE, SPC_TRA_ISO), with local optimized protocols. MRI scans of the cerebellopontine angle were included if they allowed labyrinth visualization with at least a portion of the labyrinth recognizable and suitable for manual segmentation. MRI scans, which did not allow a clear manual segmentation, were excluded from this study. In total, 259 MRI images were excluded due to unsuitable sequences (DWI, T1, SURVEY MST), poor quality, or skewed MR images. The final training dataset included MRI scans of 944 cases (Table 1).

**Table 1:** Data set characteristics.

	Training set (n = 944)	Validation set (n = 99)	Test set (n = 177)
Center (s)	Center A	Center A	Center B, C, D
Age (mean ± SD)	57.7 ± 15.9	56.7 ± 13.0	59.3 ± 14.3
Gender (M/F)	489/455	69/30	98/79
Time frame	2016–2019	2005–2015	2004–2017
Pixel spacing (mean ± SD)	0.30 ± 0.05 mm	0.29 ± 0.01 mm	0.42 ± 0.06 mm
Slice thickness (mean ± SD)	0.32 ± 0.18 mm	0.37 ± 0.06 mm	0.65 ± 0.22 mm

*N* number, *SD* standard deviation, *M* male, *F* female.

### Validation and test dataset

The validation dataset included MRI scans of 99 cases collected from Maastricht University Medical Center + (center A) in the period from 2005 to 2015 (Table 1). MRI scans collected from 3 different centers, University Hospital Antwerp (center B), Viecure Hospital Venlo (center C), and Apeldoorn dizziness center (center D) during the period of 2005–2017 (Table 1) were used as an independent Test dataset. Both validation and test dataset consisted of T2-weighted MR images of the cerebellopontine angle of patients with uni- or bilateral definitive Meniere’s disease and idiopathic asymmetric sensorineural hearing loss.

### Manual segmentation

**Figure 2:**
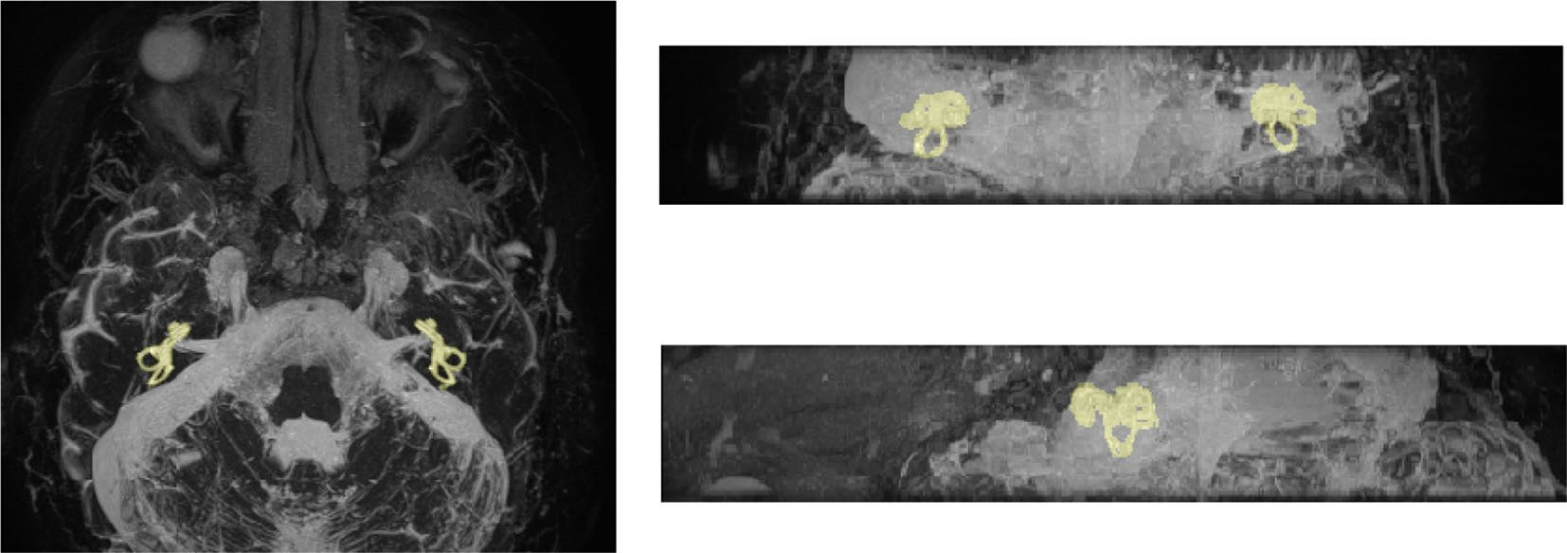
Maximum intensity projection of a sample MR in the axial, sagittal and coronal plane showing a manual segmentation of the labyrinth in yellow. Left: axial plane, right top: Coronal plane, right bottom: sagittal plane.

A team of six readers was trained by the second author (MvdL), an experienced clinician and researcher in inner ear imaging, to manually segment the labyrinth on both sides in 3D Slicer 4.8.1^19^. Manual segmentation was facilitated by intensity-based thresholding and region-growing algorithms. The original MRI scans and the manually segmented masks were visualized by 3D maximum intensity projections as shown in Fig. 2. This provided an overview of the manually segmented results which allowed for thorough quality assessment. All segmentations were curated by the experienced reader (MvdL), where any missing or incorrectly segmented masks were re-segmented by the experienced reader (MvdL). The final segmentation results served as the ground truth for training the CNN. The independent validation and test datasets were segmented and curated by the experienced reader (MvdL).The resulting manual segmentations on the test dataset was used as the ground truth (reference standard).

### Pre-processing

In order to generate homogeneous MRI volumes as input for the model, the following pre-processing steps were performed. Firstly, all volumes were resampled by B-spline interpolation to an isotropic voxel size of 0.45 mm. Secondly, the intensities of the MRI volumes were normalized to range [0–1] using the minimum and maximum intensity of each volume. Lastly, since the model’s architecture required inputs of the same dimensions, a center crop of 256 × 256 × 64 pixels was obtained from the pre-processed volumes. This crop size was large enough to contain contextual information of the inner ear. Images smaller than 256 × 256 pixels in the transversal plane and 64 pixels in slice direction were padded with zeros. More details can be found in Supplementary Information Sect. 2.

### Model architecture

**Figure 3:**
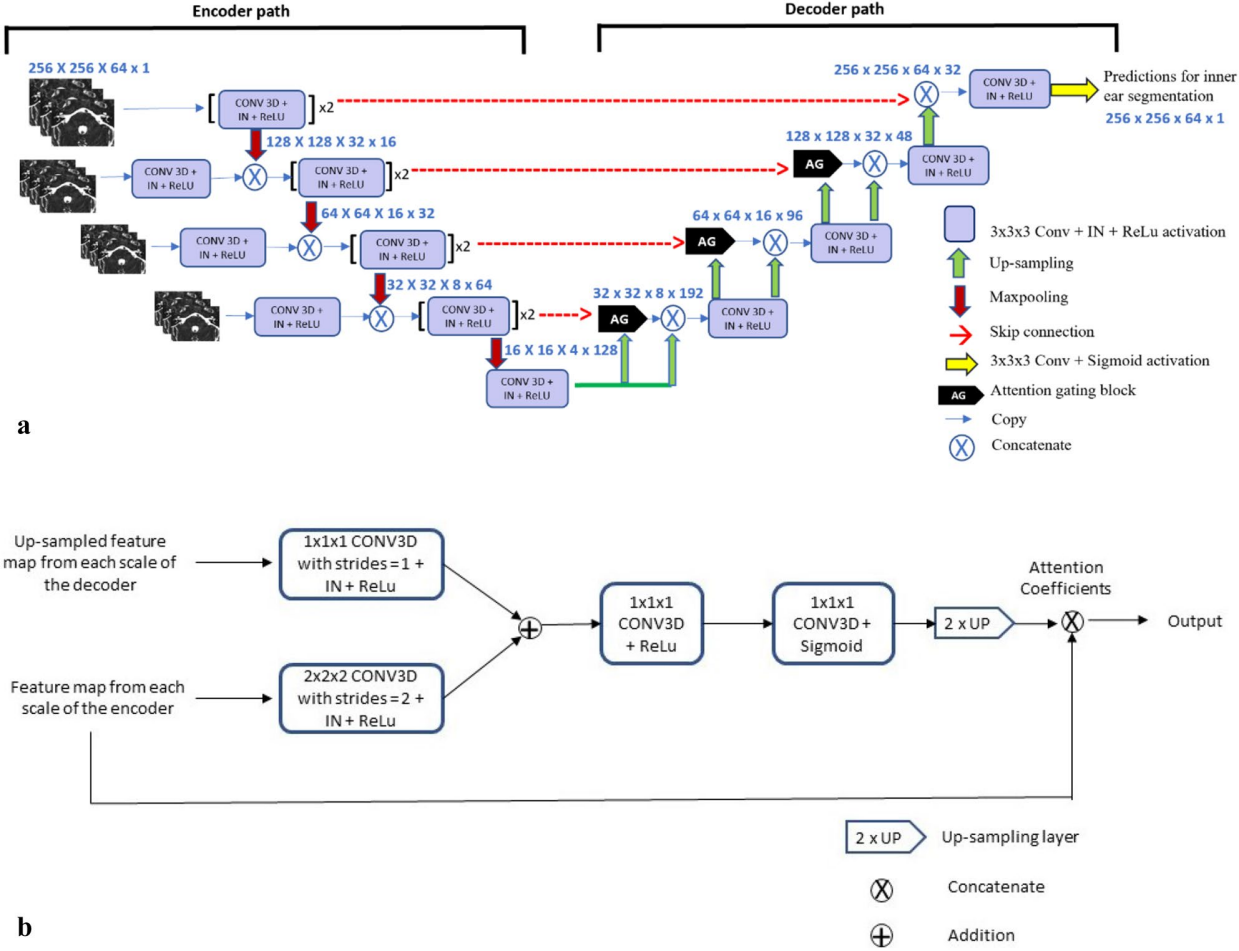
(**a**) The proposed 3D U-Net based architecture used in the study. MRI volumes, at multiple scales, were provided as input to the encoder network. The decoder network outputs a score to classify each voxel as inner ear or not. Notations in blue text (a × a × a × b) highlight the spatial resolution (a × a × a) and the feature map count (b). *X *block repetitions, *IN *instance normalization, *Conv *convolution kernel, *ReLU *rectified linear unit, *3 × 3 × 3* the size of the 3D CNN kernels. (**b**) Components of Attention Gating Block. The block receives as inputs, the up-sampled output feature map at each scale in the decoder and the feature map from each scale of the encoder. Attention coefficients generated, scale the input feature maps from the encoder.

The model’s architecture is based on a classical 3D U-net^21^, as illustrated in Fig. 3a,b. It comprises an encoder, a decoder block and skip connections. The encoder network is a contracting path with convolution layers, which extracts high-level features, decreasing the spatial resolution at each layer. The decoder network is an expanding path, which increases the spatial resolution by up-sampling and uses the feature information to segment the pixels corresponding to the Region of interest. Skip connections, between encoder and decoder, allow retrieval of fine details, which might be lost during spatial down-sampling.

The model’s architecture was adapted with attention gates, as the relevant features of the inner ear showed large shape variability and were very small compared to the surrounding structures^22^.

The attention gates highlight the regions that correspond to inner ear and suppress the regions that correspond to background. The highlighted features are propagated by the skip connections from the deep stages of contracting paths to the expanding paths. More specifically, attention Gates are used to propagate the important spatial information corresponding to inner ear from the encoding to the decoding part of the model. As shown in the Fig. 3b, the input feature maps from the encoder part of the network are scaled by the attention coefficients generated by the Attention Gates, thereby outputting the features relevant to the inner ear. The scaled features are then concatenated with the up-sampled output feature maps at each level in the decoder part of the network.

Since different components of inner ear are more easily accessible at different scales, we additionally input the same volume at three different scales along the encoder path, which has been previously described as an input image pyramid by Oktay et al*.*^22^.

Other network parameter changes included an increase in number of convolutional filters from 16 to 128 in the encoder network. Each Maxpooling layer reduced the image spatial resolution by a factor of two. Along the decoder path, transposed convolutions were used for up-sampling which increased the image size by a factor of two at each layer. All the convolutional blocks included 3D convolutions^23^, ReLu activation^24^ and Instance Normalization^25^.

### Training, validation and testing

The model was trained with the pre-processed volumes and their corresponding ground truth labels of the training dataset. Randomly selected input volumes were augmented by vertical flipping or rotation during training. The network weights were initialized by using the He-normal initialization method^26^ and updated by using the Adam optimizer^27^ at an initial learning rate of 1e^−4^.

Since the number of positive voxels (i.e. part of the inner ear) and the negative voxels were highly imbalanced, Tversky loss^28^ was used as an objective loss function while training the model, which penalized false negatives more than false positives at a false positive penalty score (β) of 0.3 and a false negative penalty score (α) of 0.7. This approach emphasizes learning features corresponding to the positive voxels. The loss was calculated in a mini batch of two images per iteration and at the end of each epoch, Tversky loss was calculated on the model’s predictions on the validation dataset to ensure validation loss convergence (i.e., decrease in validation loss).

The final model’s performance was evaluated on the multicentric, independent test dataset.

### Outcome measurements

The main outcomes of this study were the Dice similarity coefficient (DSC), true positive rate (TPR), false positive rate (FPR), false negative rate (FNR) and false discovery rate (FDR).

As a secondary outcome a subjective evaluation of clinical validation was performed by the second author (MvdL) in consensus with an experienced neuroradiologist (A.A.Postma). Towards clinical implementation, it is critical that a deep learning model is able to segment the inner ear under all conditions, including those that might alter the shape of the inner ear (e.g., by pathology). Therefore, eight MR images, with their corresponding masks, were selected by the second author (MvdL) in which the signal intensities of the inner ear were altered either by pathology or post-therapeutic changes. These scans were left out from the training dataset and were used for clinical validation of the performance of the model.

### Qualitative assessment: in silico clinical study

**Figure 4:**
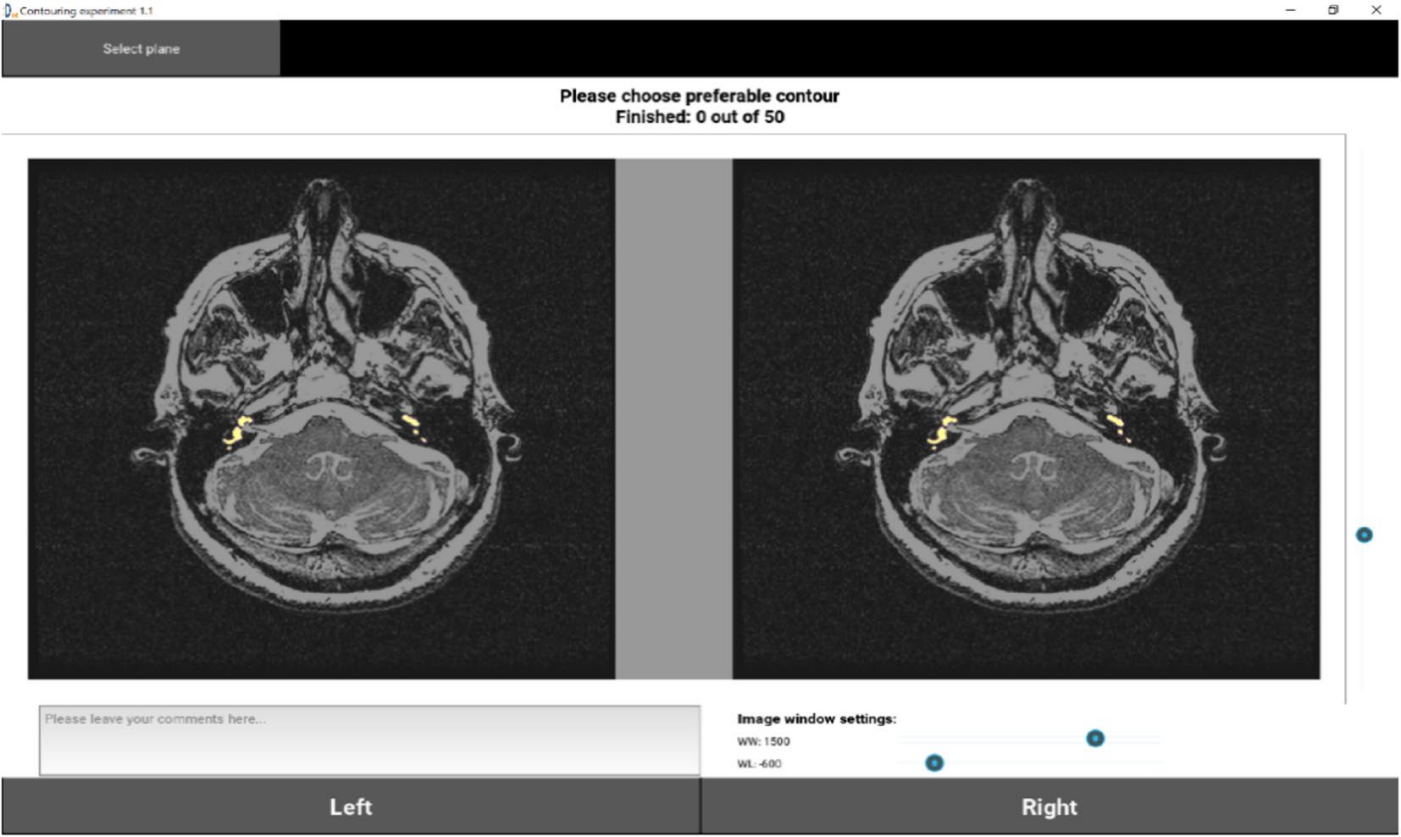
Example automated and manual segmentation overlaid on MRI volume as displayed by the software.

An in silico clinical study was performed to make a qualitative comparison between manual and model-generated segmentations for 50 MRI volumes randomly selected from the test cohort. An in-house developed software was used to display pairs of segmentations (automated vs manual), at randomized screen positions (left or right) blinded to the participants, overlaid on MRI images, as shown in Fig. 4. The software allowed for scrolling through all image slices and adjustment of window level settings. We enrolled 7 participants (3 computer scientists working in the field of medical imaging and 4 radiologists with an average experience of 2.5 years). For each image, the participants were asked to select their preferred segmentation. For each participant, the qualitative preference score was defined as the percentage of cases with preferred automated segmentation.

## Results

The final training dataset included MRI scans of 944 cases (489 men, 455 women, aged 41–74; mean age 57 ± 15). The final validation dataset included MRI scans of 99 cases from center A (69 men, 30 woman, aged 43–69; mean age 56 ± 13) and 177 cases from centers B, C and D (79 men, 59 women, aged 45–74; mean age 59 ± 14).

### Segmentation performance

**Figure 5:**
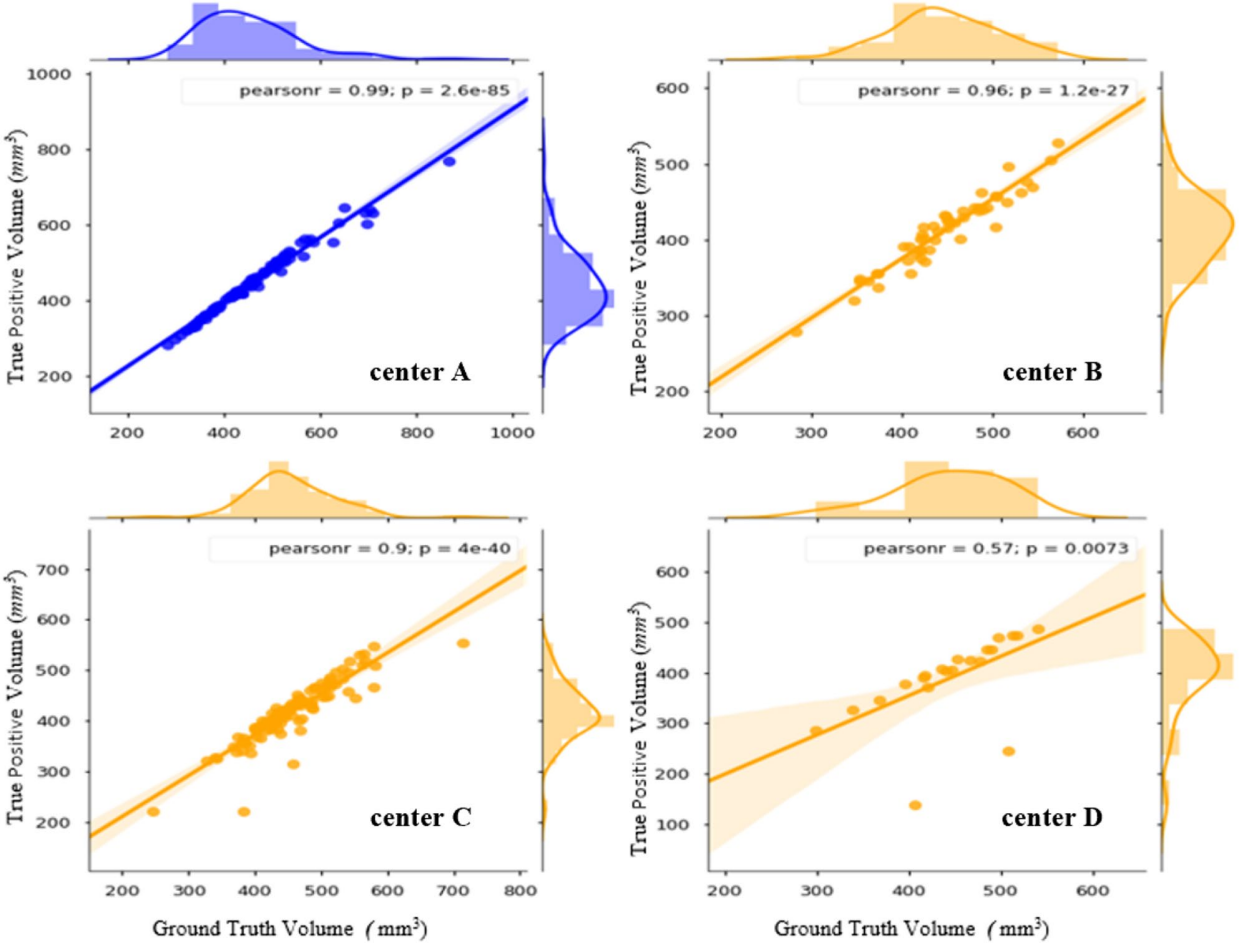
The quantitative analysis showing linear correlations between the ground truth volume and the predicted true positive volume for the validation (plot in blue) and the test sets (plots in orange). The plot of center D shows 2 clear outliers which do not fit the trendline. This suggests under-segmentation of the inner ear in 2 cases belonging to the test cohort from Center D.

**Figure 6:**
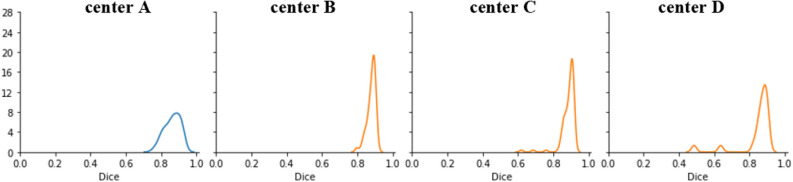
Distribution of DSC on the validation (blue curve) and the test dataset (orange curve). The distribution corresponding to Center C and D shows outliers (DSC < 0.7) which means less overlap between ground truth and predicted segmentation. The distribution also shows that the majority of the predictions have DSC between 0.8 to 1.0.

**Figure 7:**
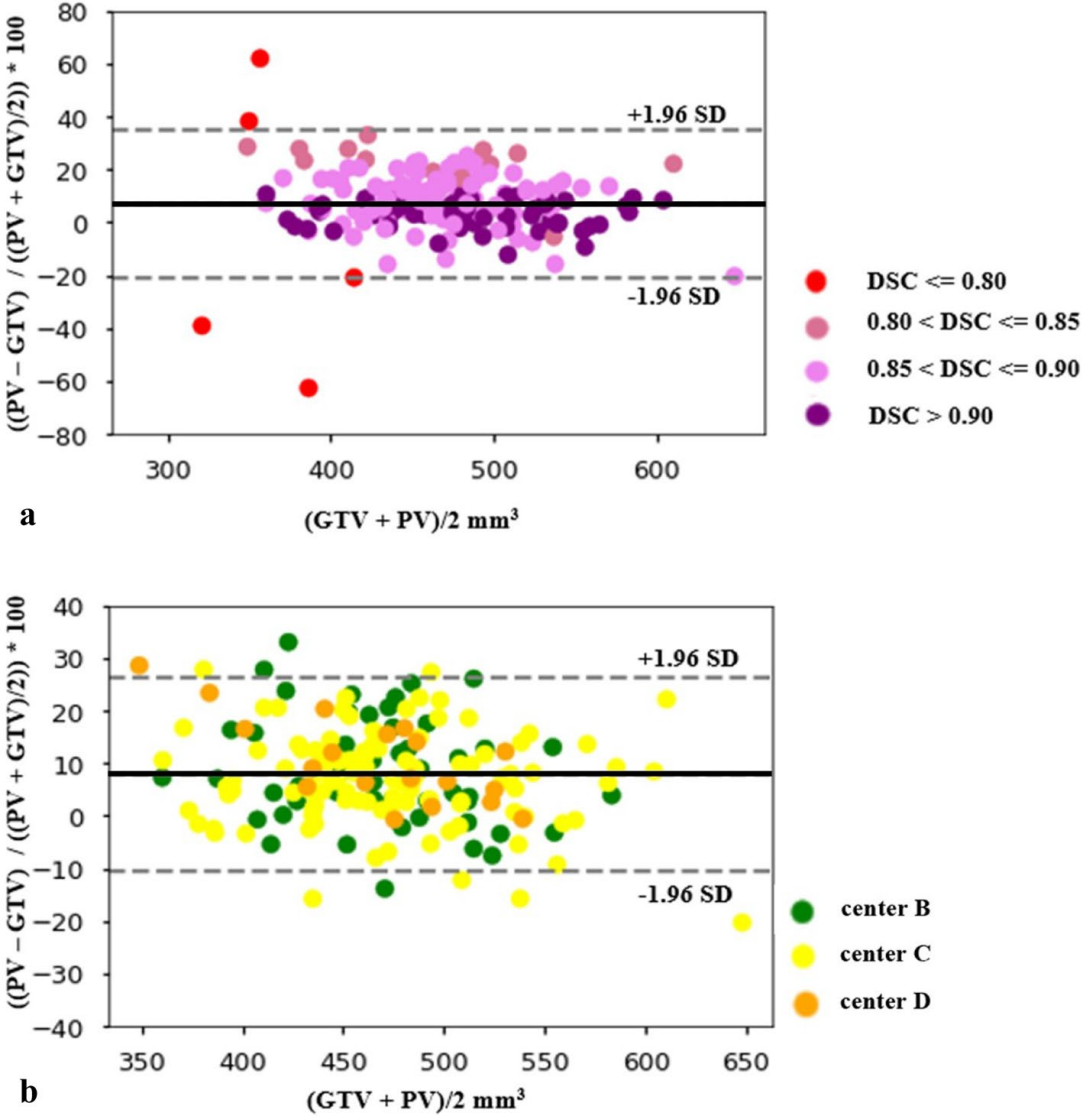
(**a**) Bland–Altman plot for inner ear volume of the entire test cohort showing percentage difference between predicted volume (PV) and ground truth volume (GTV) as a function of average of Ground Truth and Predicted Volume. The solid line shows the mean difference and the dotted line shows the limits of agreement. *PV *Predicted volume of inner ear, *GTV *ground truth volume of inner ear. The plot shows five clear outliers (red dots) with three cases which were under-segmented by 20%, 40% and 60% and two cases which were over-segmented by 40% and 60% respectively. The plot also shows the relationship between the DSC metrics and the level of under/over segmentation percentage. The outliers correspond to the DSC ≤ 0.80. (**b**) Bland–Altman plot for inner ear volume of the entire test cohort showing percentage difference between predicted volume (PV) and ground truth volume (GTV) as a function of average of ground truth and predicted volume after excluding the outliers shown in Fig. 5A (DSC ≤ 0.80). The solid line shows the mean difference and the dotted line shows the limits of agreement. *PV *Predicted volume of inner ear, *GTV *ground truth volume of inner ear. The plot shows that the model, on an average tends to over-segment by 9%.

**Figure 8:**
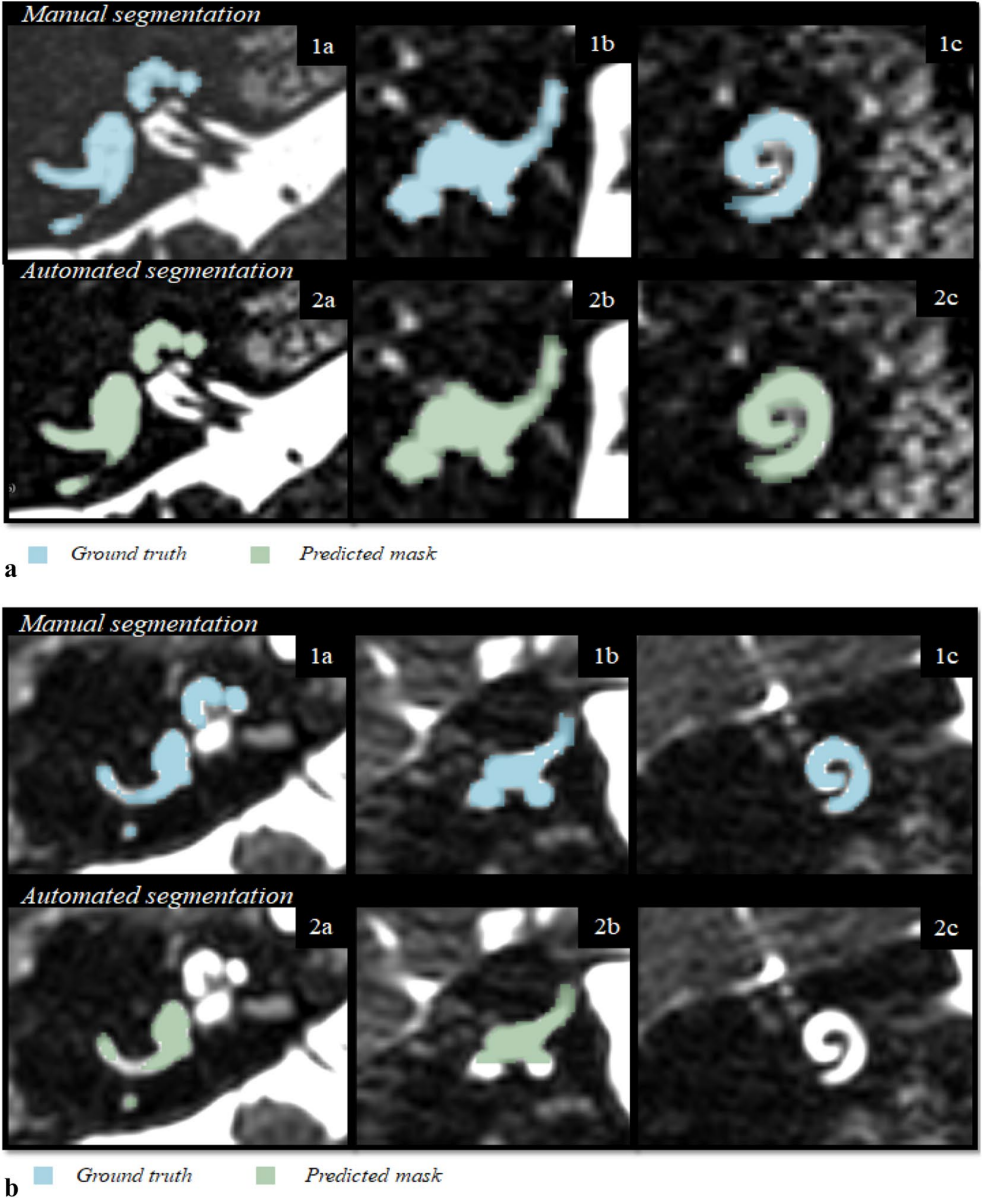
(**a**) Example of a well predicted segmentation. The first row denotes the ground truth segmentation. The second row contains the model’s segmentation. (1a) Ground truth, axial plane. (1b) Ground truth, sagittal plane. (2c) Ground truth, coronal plane. (2a) Predicted mask, axial plane. (2b) Predicted mask, sagittal plane. (2c) Predicted mask, coronal plane. DSC: 0.92, ground truth volume: 465.37 mm^3^, true positive volume: 445.32 mm^3^, true positive Rate: 95.69%, false negative rate: 4.3%. False discovery rate: 11.7%. (**b**) Example of a poor segmentation. The first row denotes the ground truth segmentation. The second row contains the model’s segmentation. (1a) Ground truth, axial plane. (1b) Ground truth, sagittal plane. (2c) Ground truth, coronal plane. (2a) Predicted mask, axial plane. (2b) Predicted mask, sagittal plane. (2c) Predicted mask, coronal plane. DSC: 0.48, ground truth volume: 406.05 mm^3^, true positive volume: 137.96 mm^3^, true positive rate: 33.97%, false negative rate: 66.02%. False discovery rate: 1.5%.

The segmentation accuracy was evaluated against the ground truth by assessing the DSC. DSC measures the overlap between the reference and the model’s output. The overall average metrics of segmentation accuracy, DSC, TPR, FNR, FDR and FPR are summarized in Table 2. Figure 5 shows a comparison between ground truth volume and predicted true positive volume on the validation and test dataset. Figure 6 shows the distribution of DSCs on validation and test dataset. The correlation between the true positive volume and ground truth volume was also investigated. In Fig. 7a,b, agreements for ground truth volume and predicted volume are graphically displayed by Bland–Altman plots. Figure 8a,b shows an example of a well predicted and poorly predicted segmentation.

**Table 2:** Performance of the proposed 3D U-Net for the automatic segmentation of the inner ear.

	Validation cohort	Test cohort
Manual vs. fully automated	99 vs. 99	177 vs. 177
DSC	0.86 (CI 0.85–087)	0.87 (CI 0.87–0.88)
True positive volume (mm^3^)	441 (CI 424–459)	412 (CI 403–421)
False positive volume (mm^3^)	123 (CI 113–134)	72 (CI 67–76)
False negative volume (mm^3^)	12 (CI 8–16)	39 (CI 34–44)
True positive rate (%)	97.7 (CI 97.2–98.3)	91.50 (CI 90–92.5)
False discovery rate (%)	21.8 (CI 21.3–22.2)	14.8 (CI 14.2–15.4)
False negative rate (%)	2.2 (CI 1.6–2.7)	8.5 (CI 7.4–9.6)

True positive volume: the volume correctly segmented as the inner ear, false negative volume: the volume incorrectly not segmented as the inner ear (under segmentation). False positive volume: the volume incorrectly segmented outside the inner ear (over segmentation) true positive rate: the percentage of voxels correctly segmented as the inner ear, false discovery rate: the percentage of voxels incorrectly segmented outside the inner ear (over segmentation), false negative rate: the percentage of voxels incorrectly not segmented as the inner ear (under segmentation).

*CI* 95% confidence interval.

### Performance on clinical validation dataset

**Figure 9:**
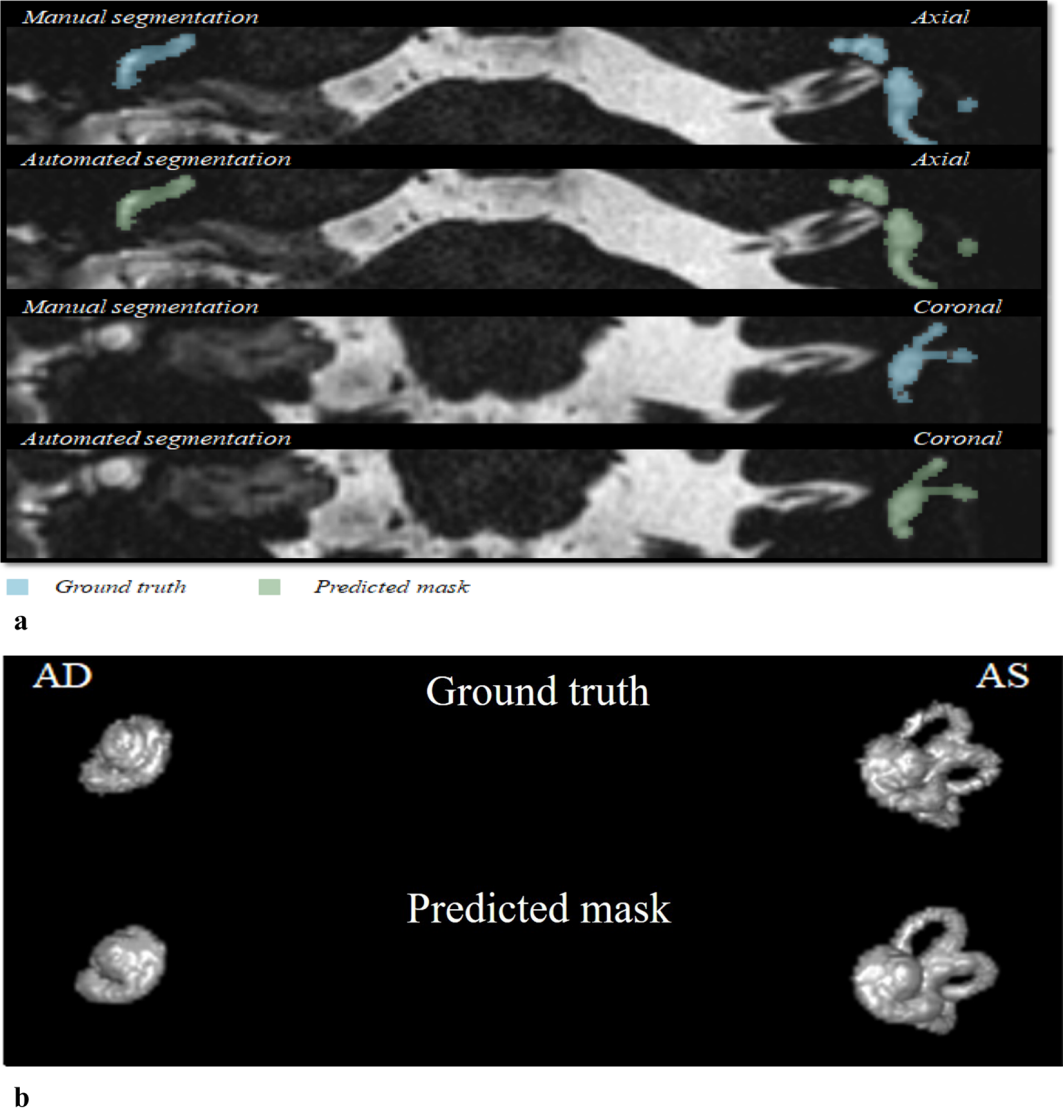
(**a**) Example of one of the clinical validation MRI scans in the axial and coronal plane. This case shows the presence of a vestibular schwannoma after a translabyrinthine resection on the right side. Therefore, the right semi-circular canals and vestibule are not segmented. DSC: 0.8973, ground truth volume: 316.11 mm^3^, true positive volume: 294.69 mm^3^, true positive rate: 93.22%, false negative rate: 6.77%. False discovery rate: 7.3%. (**b**) The 3D volume rendering of the ground truth and the predicted mask. The semi-circular canals and the vestibule of the right inner ear were not displayed on MRI. The model has correctly not segmented the semi-circular canals and the vestibule. *AD *auriculum dextra, *AS *auriculum sinistra.

On the held-out clinical validation dataset, the model achieved an average DSC of 0.876, TPR of 87.86%, FDR of 15.2% and FNR of 12.13%. The automated segmentations on this dataset are included in Supplementary Information. It includes labyrinths in which parts of the semi-circular canals, the vestibule or the cochlea were missing or not properly displayed. As an example, an MRI scan with a vestibular schwannoma (a tumorous process growing from the vestibular nerve) was included in Fig. 9.

### Qualitative assessment: in silico clinical study

On average, the participants preferred the automated segmentation in 67% of the cases. A paired one-sided t-test for the hypothesis that this average score is greater than 50% was significant (p = 9.82474e^−17^), indicating that expert users preferred the segmentations generated by the proposed model over the manual segmentations.

## Discussion

In this work, a first proof-of-concept of an artificial intelligence based model for the fully automatic segmentation of the inner ear on MRI was demonstrated and validated.

The proposed model showed high performance, with a mean DSC of 0.87 between the manual and the automated segmentation validated across images from three different centers. The mean TPR of 91.5% implies accurate segmentation of the inner ear without significant over or under segmentation as indicated by the low FDR and FNR metrics (14.8% and 8.49% respectively).

The in silico based qualitative analysis showed that on an average, the expert users (radiologists and computer scientists) are more likely to prefer model generated segmentations over manual segmentations. The Bland–Altman plot (Fig. 6) shows 5 outliers. The fact that the slice thicknesses of those scans were high (mean slice thickness—1.1 mm) compared to the mean slice thickness of the training cohort (0.32 mm) could explain the miss-segmentations for these cases. Also, the three scans from the Center D contained either moving artifacts or had tight margins around the labyrinth, which might explain the lower limits of agreements. All the MRI scans of the Center C had noticeably more hyperintense areas at the apex of the pas petrosa compared to the other centers. Although this might have ‘challenged’ the model, it does not explain why only two out of 21 scans had lower DSCs.

A prior study, that used deep learning to facilitate the auto-segmentation of the inner ear, compared the performance of a 3D Fully Connected Network (FCN) to a 2D-FCN^20^. The study reported an overall DSC of 0.66 and 0.58 when using 3D-FCN and 2D-FCN, respectively. Another recent study reported a high DSC of 0.95 using SSMs based level set^10^. However, their model was evaluated on a small dataset (10 cases out of 23 cases were held out for testing) and no independent validation was performed. Directly comparing the present approach with the already published methods in terms of DSC is not possible due to differences in datasets. Nevertheless, it is worth noting that our presented method achieves state of the art performance, which can be ascribed to the robust deep learning approach combined with a wide and varied dataset, both for training and validation, an aspect often neglected in similar studies.

There are several important strengths of this study. First of all, the model was trained on a diverse set of MR images of the cerebellopontine region. Although all MR images of the training dataset were collected in one center, they were acquired over a wide time span (2015–2019) and include different acquisition and reconstruction protocols^29^. Next to this, the training dataset was manually segmented by five independent readers. Therefore, the model learned to eliminate noise in the manually segmented labels caused by inter-reader variability. These methodological aspects resulted in a model that is well generalizable, which is reflected in the high-validation performance. Past studies have shown high inter-reader and intra-reader variability on medical image segmentation tasks^30,31^. Our method’s consistency (i.e., no segmentation variability) alleviates this issue. Additionally, the interaction time was approximately 10 min per case for manual segmentation by an experienced reader compared to only 6.5 s for the automatic segmentation.

One of the most important strengths of this study is the evaluation on the MR images containing deviant morphological shapes and decreased signal intensities of the labyrinth caused by cerebellopontine pathology. On this held-out clinical validation dataset, the model proved to generalize well with an average DSC and TPR of 0.8768 and 87.86% respectively. So far, previous auto-segmentation studies have trained their models on normal ears or small datasets (13–15). To the best of our knowledge, our study is the first to assess generalizability with respect to pathologies.

### Limitations

Several limitations of this study should be noted. First of all, the most important limitation is the lack of a gold standard for manual segmentations from highly experienced neuroradiologists. Due to the extent of the segmentation process, manual segmentation of approximately 1500 labyrinths by one or more senior radiologist was not feasible. Therefore, in this study the authors chose to work with independent readers who were trained and supervised by an experienced clinical researcher in inner ear imaging (MvdL) to generate a first proof of concept. This could have induced noise in the manual segmentations. Also, the intra- and inter-observer variability of the segmentation team was not evaluated. Although manual segmentation was performed under strict supervision of the second author and a curating process was performed to detect incorrectly segmented masks, the quality of the manual segmentation could not be fully guaranteed. Since the manually segmented masks were considered as the reference standard for the evaluation of the model, lower DSC scores might have indicated better automated segmentation compared to manual segmentation.

Nevertheless, efforts have been made to contain this limitation by training a deep learning architecture with large number of parameters and applying Early Stopping to prevent overfitting on the noise in the manual segmentation. Previous studies have proved that overparameterized networks are more robust against noisy labels when Early Stopping is applied^32^.

Given the very small are occupied by the inner ear in the whole MRI volume, the performance of our model might be further improved by applying bounding box detection^33^ or shape identification^34^ prior to automated segmentation especially for abnormal cases.

Secondly, poor generalizability is the most common problems pertaining to deep learning models^29^. In this study, attempts were made to prevent overfitting by training the model on a large dataset from one center and testing its generalizability by holding out 3 independent validation cohorts. Although the overall DSC scores were markedly high, the model performed poorly and failed to generalize in five cases out of 177 (3 cases from center C and 2 cases from center D had DSC < 0.70). This situation could have been mitigated by training the model on all of the centers. This would have made the training dataset more diverse (e.g., in terms of image acquisition and reconstruction) and the model’s performance could have been evaluated by cross-validation techniques (i.e., holding out 20–30% of the data from each center for a single validation test data). However, this would degrade the credibility of the generalizability of the model due to concerns regarding overfitting.

Lastly, the model was trained and evaluated on datasets that included only Dutch and Belgian population. The generalizability of the model on MRI images from an international cohort is currently unexplored.

### Clinical implications and future perspectives

The future clinical advantages of automated 3D image segmentation of the inner ear are versatile. Image segmentation can be used for 3D visualization, allowing a better understanding of the spatial relations and morphological changes within the inner ear, assisting radiologists in the diagnostic process and providing tools for surgical planning^35^ or learning purposes^36^. Previous studies have proven the usability of auto-segmentation for pre-operative planning of cochlear implant surgery using CT imaging^37^ and for the diagnosis of adolescent idiopathic scoliosis using MRI imaging^11^. Our model proved to be efficient on MRI imaging. However, the proposed methodology can be easily leveraged for similar auto-segmentation applications on different imaging modalities.

Nowadays, quantitative analysis of the inner ear is gaining more importance. Techniques like radiomics^6^, volumetric assessment of fluid compartments in the labyrinth^12,38^ and the analysis of the morphoanatomy for the vestibular system^11^ are used to aid diagnosis of vestibular diseases. *Radiomics* refers to the process of the automated extraction and analysis of large amounts of quantitative features from medical images. These features are sometimes not perceptual for the human eye and might contain information that reflects underlying tissue heterogeneity and pathophysiology^4,39^. Quantitative image features involve descriptors of shape, size, volume, intensity distributions and texture heterogeneity patterns^39^.

A histological feature strongly associated with Meniere’s disease is endolymphatic hydrops (EH), a distention of the endolymphatic compartment in the inner ear^40^. In conventional MRI, the endolymphatic compartment cannot be distinguished from the perilymphatic compartment, and thus, EH is not depicted^41^. The differences found in radiomic features between MD and controls could hypothetically be explained by the different composition of the fluids in the labyrinth, causing a different distribution of signal intensities^5^. Possibly, EH is captured in the quantitative image features due to damage to or morphological changes to the endolymphatic space. Since Meniere’s disease is still a clinical diagnosis challenge^42^, discovering distinctive image features might benefit the diagnostic trajectory of MD. Another study showed that cochlea CT image features can be useful biomarkers for predicting sensorineural hearing loss in patient with head and neck cancers which received chemoradiation therapy^6^. Different machine learning methods were used for feature selection, classification and prediction. The advantage of using machine learning in combination with radiomics is that the analysis of the labyrinth could be done autonomously in the future^5^. However, for both studies, setting a Region Of Interest (ROI) by manual segmentation was necessary. The fully automated segmentation of the inner ear contributes to efficient research on quantitative image analysis of the inner ear.

Next to analyses on conventional MRI and CT imaging, the volumetric assessment of fluid compartments in the labyrinth is also promising for vestibular research^38^. Contrast-enhanced MR imaging allows the in vivo confirmation and quantification of endolymphatic hydrops^12,43^.

Several studies investigated the value of the 3D volumetric assessment of the endolymphatic space (ELS) to better monitor EH in vivo, for example in therapeutic trials in Meniere’s disease, and to better compare the ELS in patients with different otological diseases^38,44,45^. However, the 3D reconstruction were all rendered semi-automatic. Due to this time-consuming process, the applications for volumetric assessment are yet more scientifically than clinically relevant. A recent study proposed atlas-based segmentation for the volume-based quantification of the fluid spaces of the inner ear^12^. Which created fast, standardized (auto)segmentation. Further research is necessary to explore the option of the proposed U-net model can be leveraged for contrast-enhanced imaging as well, to facilitate volumetric assessment of the ELS in clinic.

Auto-segmentation in its current form, is a step towards fully automated diagnostic tools for inner ear disorders.

## Conclusion

In this study, a working first proof-of-concept is demonstrated regarding the fully automatic segmentation of the inner ear using deep learning. Overall, the proposed auto-segmentation model is equivalent to manual segmentation and is a reliable, consistent, and efficient method for inner ear segmentation which can be used in a variety of clinical applications, such as 3D visualization, surgical planning and quantitative image analysis. Auto-segmentation of the inner ear in its current form, might open doors towards automated diagnostic tools for inner ear disorders.

## Supplementary Information

Supplementary Information.

## Abbreviations

DSC: Dice similarity coefficient
TPR: True positive rate
FNR: False negative rate
FPR: False positive rate
FDR: False discovery rate
