# Supplementary material for: Deep learning for the fully automated segmentation of the inner ear on MRI
Source: Sci Rep. 2021 Feb 3;11:2885. doi: 10.1038/s41598-021-82289-y (PMC7858625; doi:10.1038/s41598-021-82289-y)
Supplement: Supplementary file 1 — Supplementary Information. [file 41598_2021_82289_MOESM1_ESM.docx]

***Supplementary Materials***

**Title**

Deep learning for the fully automated segmentation of the inner ear on MRI

**Authors**

Akshayaa Vaidyanathan^a,b *^ , Marly F.J.A van der Lubbe^c^ , Ralph T.H. Leijenaar^b^ , Marc van Hoof^c^ , Fadila Zerka^b^, Benjamin Miraglio^b^, Sergey Primakov^a^ , Alida A. Postma^d,e^ , Tjasse .D. Bruintjes^f^ , Monique A.L. Bilderbeek^g^, Hammer Sebastiaan^k^, Patrick F.M. Dammeijer^h^, Vincent van Rompaey ^i^ , Henry C. Woodruff^a,j^, Wim Vos^b^, Seán Walsh^b^, Raymond van de Berg^c,e ‡^, Philippe Lambin^a,j ‡^

‡Raymond van de Berg & Philippe Lambin have equally contributed as last author.

**Author information**

1. The D-Lab, Department of Precision Medicine, GROW - School for Oncology, Maastricht University, Maastricht, NL, Department of Radiology and Nuclear Imaging, GROW- School for Oncology, Maastricht University Medical Centre, Maastricht, NL.
2. Oncoradiomics SA, Liege, Belgium
3. Department of Otolaryngology and Head and Neck Surgery, Maastricht University Medical Center, Maastricht, The Netherlands.
4. Department of Radiology and Nuclear Medicine, Maastricht University Medical Center, Maastricht, The Netherlands
5. School for Mental Health and Sciences, Maastricht University, Maastricht, The Netherlands
6. Department of Otorhinolaryngology, Gelre Hospital, Apeldoorn, The Netherlands, Department of Otorhinolaryngology, Leiden University Medical Center, Leiden NL
7. Department of Radiology, Viecuri Medical Center, Venlo, The Netherlands
8. Department of Otorhinolaryngology, Viecuri Medical Center, Venlo, The Netherlands
9. Department of Otorhinolaryngology and Head & Neck Surgery, Antwerp University Hospital; Dept. Translational Neuroscience, Faculty of Medicine and Health Sciences, University of Antwerp, Antwerp, Belgium
10. Department of Radiology and Nuclear Medicine, GROW - School for Oncology and Developmental Biology, Maastricht University Medical Centre+, Maastricht, The Netherlands
11. Haga Hospital, Radiology, Els Borst-Eilersplein 275, Den Haag, Zuid-Holland

**Corresponding author**: Akshayaa Vaidyanathan, Clos Chanmurly 13, 4000 Liège, Belgium, [akshayaa.vaidyanathan@oncoradiomics.com](mailto:akshayaa.vaidyanathan@oncoradiomics.com)

**Section 1**

Model Architecture, training and testing

First, cropped and pre-processed MRI volumes at dimensions 256 x 256 x 64 x 1 (Height X Width X number of axial slices X Channels) were processed by the two 3D convolution blocks. The 3D convolution block comprised a 3D convolution layer with 3x3 pixel filters, an instance normalization layer and a Rectified Linear Unit activation layer.

Instance normalization is one of the techniques to reduce Internal Covariate shift (1). (This normalization strategy was chosen rather than the commonly used Batch Normalization(BN), as the batch size is restricted to 2 due to memory constrains and using BN when training with such small batch size had been proven to result in noisy estimation of normalization parameters and bad convergence (2).)

Output features from the second 3D convolution block were then processed in a 3D maximum pooling layer with a kernel size 2. 3D maxpooling layer calculates maximum value among K x K x K pixels (Kernel size, K=2). This combination of two 3D convolution blocks and one 3D maxpooling layer was repeated four times. Therefore, there were 8 convolution blocks and 4 maxpooling layers. Also, the output from each Maxpooling layer was concatenated with input image at lower resolutions processed by single 3D convolutional layer as shown in Figure 2. 3D Average pooling function was applied on the original image to obtain input image at lower resolutions ([128 x 128 x 32], [64 x 64 x 16], [32 x 32 x 8]). This setup constituted the encoder path of the model where high-level semantic features were extracted.

The output from the encoder path was processed by a 3D Upsampling layer and a 3D convolution block. This combination of 3D Upsampling layer and 3D convolution block was repeated 4 times. Each upsampling layer increased the spatial resolution by a factor of 2. Also, the output from each upsampling layer was concatenated with the output from the convolution blocks in the encoder which were processed by an Attention gating block (exhibiting the functionality of skip connections in the UNet). The structure of the Attention gating block was equivalent to the implementation in the original paper (3).

The output from the last convolution block was processed by 3D convolution layer and a sigmoid activation function. Sigmoid activation layer produced output image with values for each pixel ranging from 0 to 1.

Tversky loss function (4) was used to compute error between the predicted output and the reference label (ground-truth) and the error was back-propagated using Adam Optimizer (5).

To reduce the computational cost and to be able to fit the GPU memory, groups with a small number of images (n = 2), called as mini batches, were generated after randomly shuffling the training dataset. Image augmentation (vertical flipping) was performed during training on randomly selected images.

Error calculations were performed per mini batch and parameters of the model were updated. Loss convergence on the validation dataset was considered as a criteria to check for overfitting (6) and for exhibiting early-stopping (7). Both training and validation loss converged till 9^th^ epoch and during the 10^th^ epoch, the training loss decreased while the validation loss increased drastically, hence early stopping at the 10^th^ epoch was performed to prevent overfitting on the training dataset. Weights saved at the 9^th^ epoch were used during the model evaluation.

During test phase, the output predictions from the sigmoid activation function was thresholded to 0.5 to obtain binary masks of inner ear segmentation (i,e, pixels with predictions less than 0.5 were clipped to 0 and pixels with predictions above 0.5 were clipped to 1).

**Section 2**

Pre-processing

Regarding intensity rescaling, this was performed in the following steps:

Intensities with negative values are clipped to zero and Min-max normalization:

image = (image - min_intensity) / (Max_intensity - min_intensity)

where,

Image = input image

min_intensity = minimum of the pixel intensities in the image

max_intensity = maximum of the pixel intensities in the image

Regarding Augmentation, the rotation angles ranged between 2 degrees to 15 degrees.

Rotation was performed using the rotate function from the python package scikit-image (version 0.16.1) (8)

Following values were passed as parameters to the rotate function:

Image = input image

Angle = ranged between 2 degrees to 15 degrees

Resize = False

Center = None

Order = 1

Mode = ‘constant’

Cval = 0

Preserve_range = True

The above values were passed as parameters in-order to perform the rotation around the center of the image using spline interpolation of order 1. After rotation, the points outside the boundaries of the image are filled with zeros. The range of intensities in the preprocessed image ([0,1]) is retained after rotation.

1. Molina CRR, Vila OP. Solving internal covariate shift in deep learning with linked neurons. 2017;

2. Lian X, Liu J. Revisit Batch Normalization: New Understanding and Refinement via Composition Optimization. 2019.

3. Oktay O, Schlemper J, Folgoc LL, et al. Attention U-Net: Learning Where to Look for the Pancreas. 2018;

4. Abraham N, Khan NM. A novel focal tversky loss function with improved attention u-net for lesion segmentation. Proc - Int Symp Biomed Imaging. 2019.

5. Kingma DP, Ba JL. Adam: A method for stochastic optimization. 3rd Int Conf Learn Represent ICLR 2015 - Conf Track Proc. 2015.

6. Caruana R, Lawrence S, Giles L. Overfitting in neural nets: Backpropagation, conjugate gradient, and early stopping. Adv Neural Inf Process Syst. 2001.

7. Ying X. An Overview of Overfitting and its Solutions. IOP Publishing; 2019;22022.

8. van der Walt S, Schönberger JL, Nunez-Iglesias J, et al. scikit-image: image processing in Python. Gomez S, editor. PeerJ. 2014;2:e453https://doi.org/10.7717/peerj.453.

***Supplementary figures***


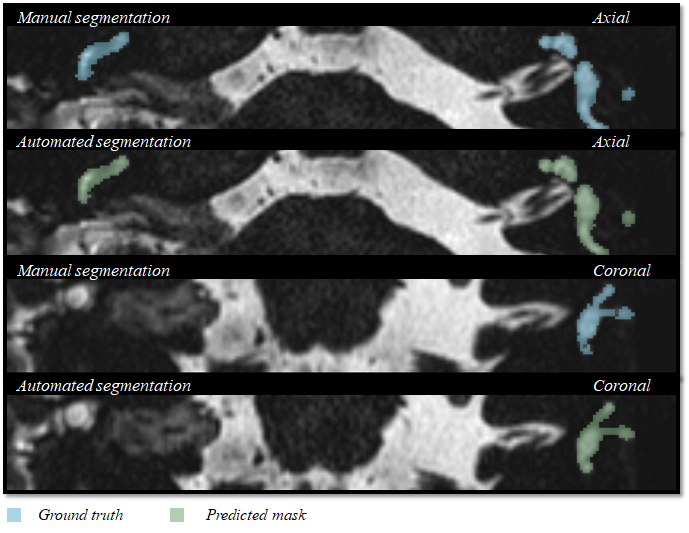


**Figure 1b**. The 3D volume rendering of the ground truth and predicted mask in anterior view. The right labyrinth ear is missing the vestibule and the semicircular canals AD= auriculum dextra, AS=auriculum sinistra


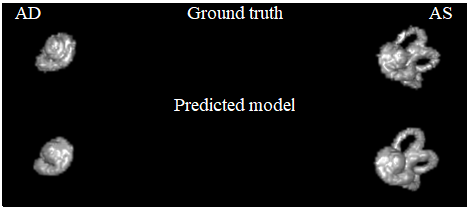


**Figure 1a.** Example of one of the clinical validation MRI scans in the axial and coronal plane. This case shows the presence of a vestibular schwannoma after a translabyrinthine resection on the right side. Therefore, the right semi-circular canals and vestibule are not segmented. DSC: 0.8973, Ground Truth Volume: 316.11 mm^3^, True Positive Volume: 294.69mm^3^, True Positive Rate: 93.22%, False Negative Rate: 6.77%. *False* Positive Rate: 0.0005%

**Figure 1b.** The 3D volume rendering of the ground truth and the predicted mask. The semi-circular canals and the vestibule of the right inner ear were not displayed on MRI. The model has correctly not segmented the semi-cicular canals and the vestibule. AD= auriculum dextra, AS=auriculum sinistra


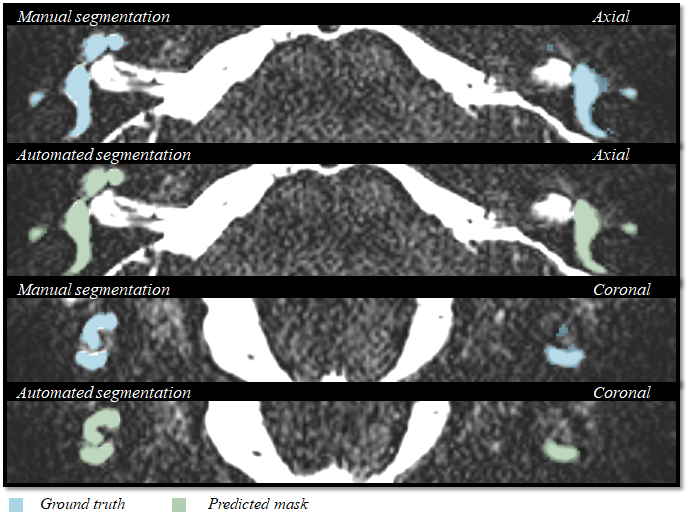


**Figure 2a.** Example of one of the clinical validation MRI scans in the axial and coronal plane. This case shows obliteration of the apical and middle turn of the left cochlea, indicating the presence of either labyrinthitis ossificans or a vestibular schwannoma. The left cochlea is, therefore, not fully segmented. DSC: 0.8691, Ground Truth Volume: 680.79 mm^3^, True Positive Volume: 573.26 mm^3^, True Positive Rate: 84.20%, False Negative Rate: 15.79%. *False* Positive Rate: 0.0007%


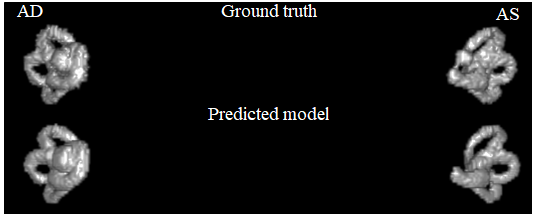


**Figure 2b.** The 3D volume rendering of the ground truth and the predicted mask. The cochlea of the left inner ear was not fully displayed on MRI. The model has correctly not segmented parts of the cochlea. AD= auriculum dextra, AS=auriculum sinistra


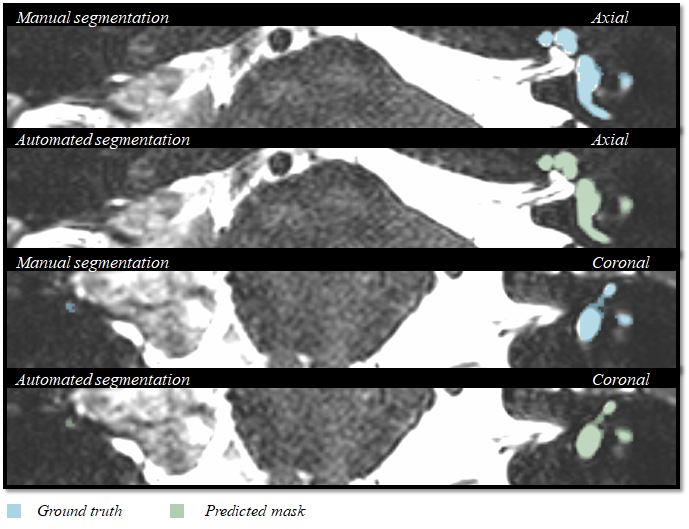


**Figure 3a.** Example of one of the clinical validation MRI scans in the axial and coronal plane. This case shows the presence of a vestibular schwannoma after a translabyrinthine resection on the right side. The left semi-circular canals and the vestibule are, therefore, not segmented. DSC: 0.8704, Ground Truth Volume: 359.94 mm^3^, True Positive Volume: 314.92 mm^3^, True Positive Rate: 87.49%, False Negative Rate: 12.50%, *False* Positive Rate: 0.0005%


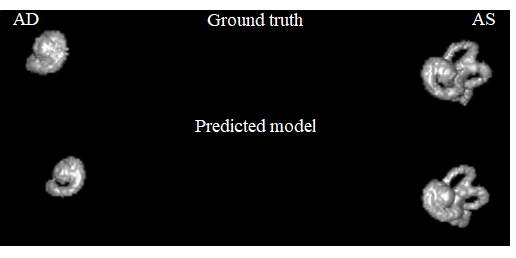


**Figure 3b** The 3D volume rendering of the ground truth and the predicted mask. The semi-circular canals and the vestibule of the right inner ear were not displayed on MRI. The model has correctly not segmented the semi-circular canals and the vestibule. AD= auriculum dextra, AS=auriculum sinistra


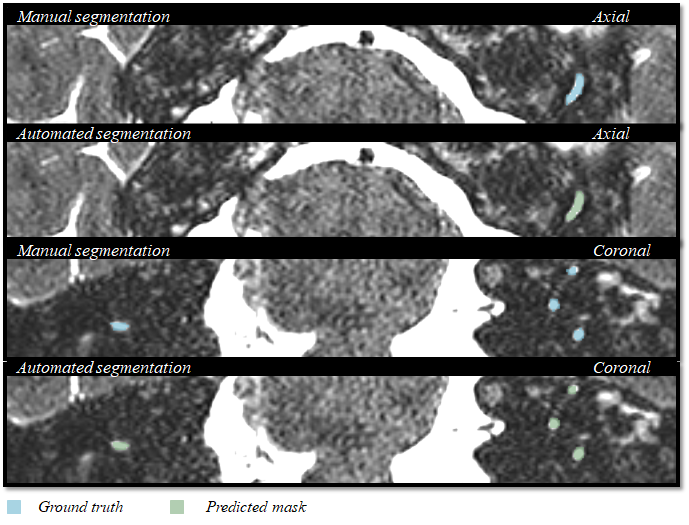


**Figure 4a.** Example of one of the clinical validation MRI scans in the axial and coronal plane. This case shows post-therapeutic fibrosis in the left inner ear. The right superior and inferior semi-circular canals are not segmented. DSC: 0.8916, Ground Truth Volume: 510.3 mm^3^, True Positive Volume: 442.5 mm^3^, True Positive Rate: 86.71%, False Negative Rate: 13.28%. *False* Positive Rate: 0.0004%


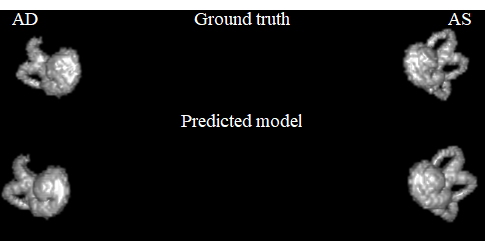


**Figure 4b.** The 3D volume rendering of the ground truth and the predicted mask. The superior and inferior semi-circular canals of the right inner ear were not displayed on MRI. The model has correctly not segmented these semi-circular canals AD= auriculum dextra, AS=auriculum sinistra


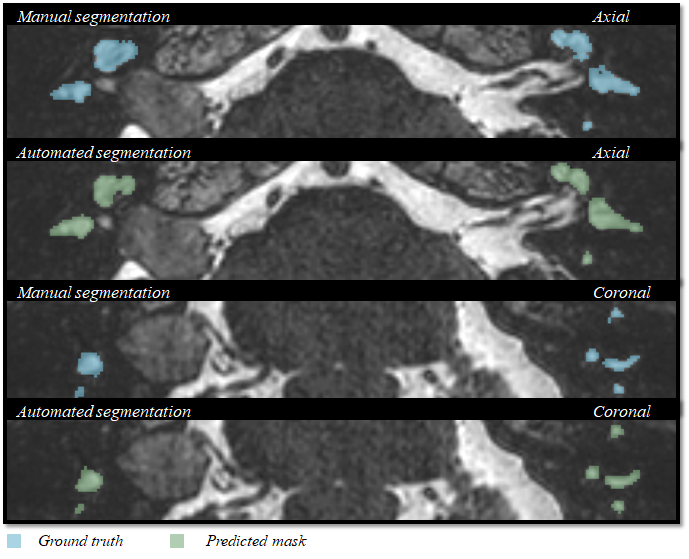


**Figure 5a.** Example of one of the clinical validation MRI scans in the axial and coronal plane. This case shows the presence of a vestibular schwannoma on the right side, with changes in the signal intensities the inner ear, indicating fibrosis. The right, superior, lateral and inferior semi-circular canals are not fully segmented. DSC: 0.8770, Ground Truth Volume: 486.78 mm^3^, True Positive Volume: 434.21 mm^3^ , True Positive Rate: 89.19%, False Negative Rate: 10.8% . *False* Positive Rate: 0.013%


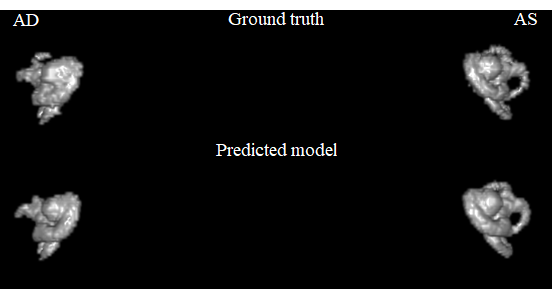


**Figure 5.B** The 3D volume rendering of the ground truth and the predicted mask. The superior, lateral and inferior semi-circular canals of the right inner ear were not properly displayed on MRI. The model has correctly not segmented these semi-circular canals AD= auriculum dextra, AS=auriculum sinistra


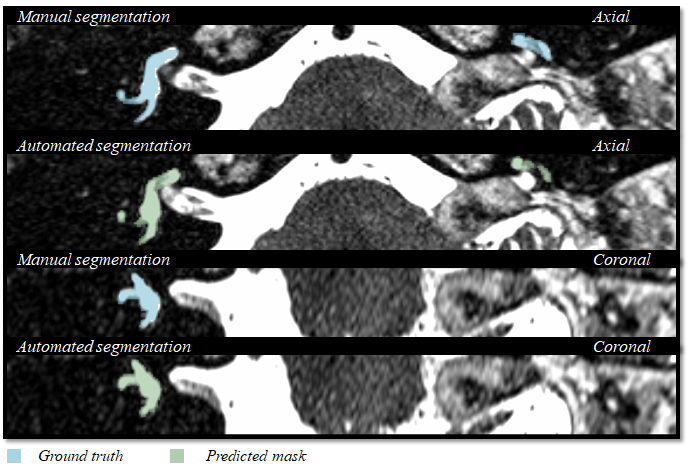


**Figure 6a.** Example of one of the clinical validation MRI scans in the axial and coronal plane. This case shows the presence of a vestibular schwannoma after a translabyrinthine resection on the left side. The left semi-circular canals and vestibule are not segmented. DSC: 0.8631, Ground Truth Volume: 395.39mm^3^, True Positive Volume: 348.55 mm^3^ , True Positive Rate: 88.15% , False Negative Rate: 11.84%, *False* Positive Rate: 0.0004%


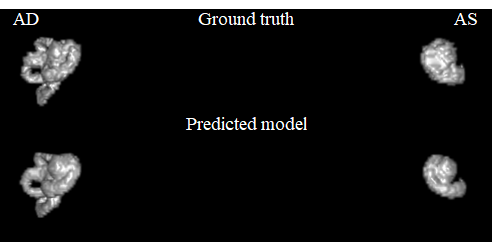


**Figure 6b.** The 3D volume rendering of the ground truth and the predicted mask. The semi-circular canals and the vestibule of the right inner ear were not displayed on MRI. The model has correctly not segmented the semi-circular canals and vestibule. AD= auriculum dextra, AS=auriculum sinistra


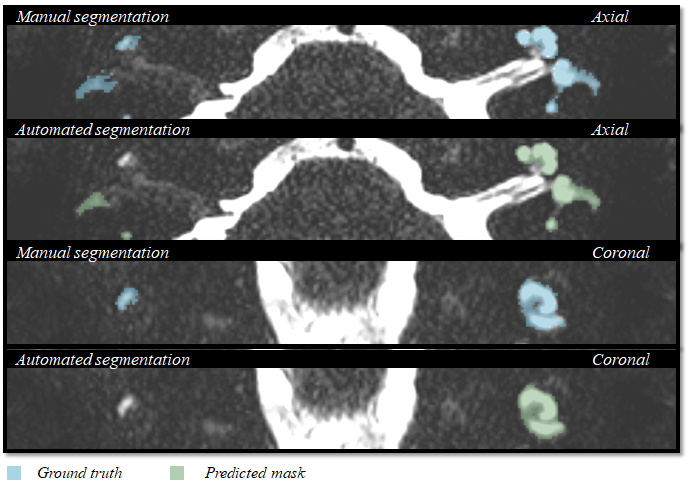


**Figure 7a.** Example of one of the clinical validation MRI scans in the axial and coronal plane This case shows a transmodiolar and macular schwannoma on the right side. The right vestibule and cochlea are not fully segmented. DSC: 0.8648, Ground Truth Volume: 472.39 mm^3^, True Positive Volume:405.14 mm^3^, True Positive Rate:85.76%, False Negative Rate:14.23% . *False* Positive Rate: 0.0006%


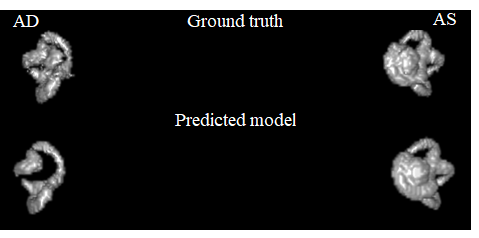


**Figure 7b.** The 3D volume rendering of the ground truth and the predicted mask. The vestibule and the cochlea of the right inner ear were not displayed on MRI. The model has correctly not segmented the vestibule and the cochlea. AD= auriculum dextra, AS=auriculum sinistra


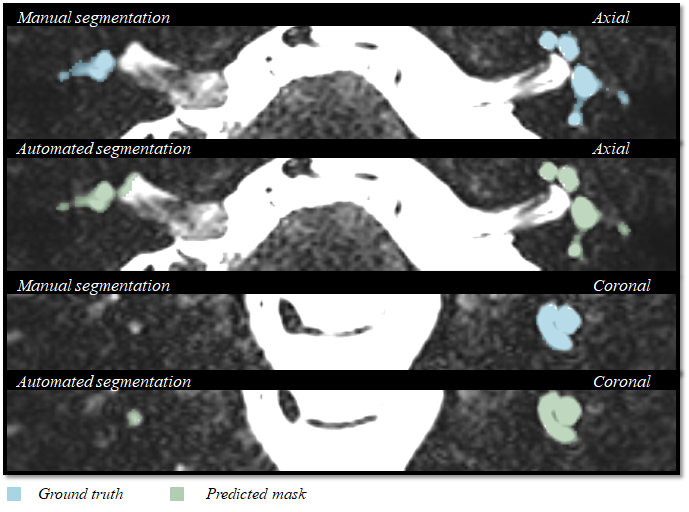


**Figure 8a.** Example of one of the clinical validation MRI scans in the axial and coronal plane. This case shows obliteration of the apical, middle and basal turn of the right cochlea, indicating the presence of either labyrinthitis ossificans or a vestibular schwannoma. The right superior and inferior semi-circular canals, the vestibule and the cochlea are not fully segmented. DSC: 0.8810, Ground Truth Volume: 369.603 mm^3^, True Positive Volume: 325.95 mm^3^ , True Positive Rate: 88.19%, False Negative Rate: 11.80%. *False* Positive Rate: 0.0005%


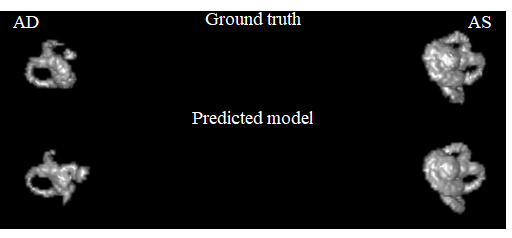


**Figure 8b.** The 3D volume rendering of the ground truth and the predicted mask. The superior and inferior semi-circular canals, the vestibule and the cochlea of the right inner ear were not displayed on MRI. The model has correctly not segmented these structures. AD= auriculum dextra, AS=auriculum sinistra
